# Screening of potential ligands from the ZINC database with RAC1B using molecular docking and dynamics simulation analysis

**DOI:** 10.6026/973206300214379

**Published:** 2025-12-15

**Authors:** Kajal Verma, Lakshmi Pillai

**Affiliations:** 1 Institute of Sciences, SAGE University, Indore, Madhya Pradesh, India

**Keywords:** Breast cancer, RAC1B, pharmacophore modelling, molecular docking, molecular dynamics simulation, ADME

## Abstract

Breast cancer remains the most frequently diagnosed cancer among women worldwide. Therefore, it is of interest to document the Screening for potential ligands from the ZINC database with RAC1B using molecular docking and dynamics simulation analysis. Data shows the optimal binding of five lead compounds ZINC5277811, ZINC16485670, ZINC20530618, ZINC29990274, and ZINC3528881 with RAC1B for further consideration.

## Background:

In 2020, 2.3 million women were diagnosed with breast cancer (BC) with 685,000 deaths globally [1]. At the end of 2020, 7.8 million were diagnosed with BC in the past 5 years, making BC the world's most prevalent cancer [2]. The estimated new total BC cases in the United States (US) for 2022 are 287,750 for females and 2710 for males, respectively [3]. The unchecked division or proliferation of breast cells results in breast cancer [4]. A 1600 BC Egyptian study on women's history revealed that milk, which is linked to the initial form of breast cancer, is supplied by the inner layers of milk ducts [5]. Although men can contract it as well, women are more likely to contract this illness in wealthy nations [6]. Breast cancer ranks second globally in terms of cancer incidence and it is a major problem [7]. In 2011, 508,000 women lost their lives to breast cancer and in 2012, there were 1.7 million new cases of the disease [8]. According to estimates from the American Cancer Society, 246,660 new instances of invasive breast cancer were detected in American women in 2016 [9]. Age, heredity, height, radiation exposure, smoking, obesity, alcohol use, being overweight and a dislike of nursing are the primary causes of this malignancy [10]. There are several proteins involved in breast cancer but I chose RAC1B since this protein is also implicated in many other cancers [11].

RAC1B, a lesser-known isoform of the protein RAC1, has emerged as a potential key player in breast cancer development and progression [12]. RAC1B promotes cell migration and invasion, key steps in tumor metastasis [13]. It activates signalling pathways that regulate cell movement and cytoskeletal reorganization [14]. This allows cancer cells to detach from the primary tumors, travel through the bloodstream and colonize other organs [15]. RAC1B overexpression is linked to resistance to chemotherapy drugs like doxorubicin [16]. It may activate survival pathways that protect cancer cells from drug-induced cell death [17]. Inhibiting RAC1B could improve the effectiveness of chemotherapy and overcome treatment resistance [18]. RAC1B'S specific role in CSCs and resistance makes it a promising target for developing new cancer therapies [19]. Detecting high RAC1B levels in tumors could aid diagnosis and predict treatment response [20]. Drugs or therapies specifically targeting RAC1B could be used to eliminate CSCs prevent metastasis and improve treatment efficacy [21]. Overall, RAC1B research holds significant promise for improving our understanding and treatment of breast cancer [22]. Targeting this protein may offer new strategies to suppress tumor growth and metastasis [23]. RAC1B was recently identified in malignant colorectal tumors as an alternative splice variant of Rac1 containing a 19-amino acid insertion next to the switch II region [24]. The structures of RAC1B in the GDP- and the GppNHp-bound forms, determined at a resolution of 1.75 A, reveal that the insertion induces an open switch I conformation and a highly mobile switch II [25]. As a consequence, RAC1B has an accelerated GEF-independent GDP/GTP exchange and an impaired GTP hydrolysis, which is restored partially by GTPase-activating proteins [26]. Interestingly, RAC1B can bind the GTPase-binding domain of PAK but not full-length PAK in a GTP-dependent manner, suggesting that the insertion does not completely abolish effector interaction [27]. RAC1B has an additional exon (i.e., exon3b) encoding 19 amino acids with an in-frame insertion just after its Switch-II domain [28]. This leads to a structural change favouring the active GTP-bound state independent of GEF-mediated activation [29]. RAC1B function is essential in BCSCs for their plasticity, chemo resistance to doxorubicin treatment and tumor-initiating abilities [30]. Molecular docking analysis of breast cancer target RAC1B with ligands from drug database is shown [31]. Hence, immediate attention is needed to find potent inhibitors against RAC1B involved in breast cancer [32]. Followed by Absorption, distribution, metabolism, excretion (ADME) property prediction, and molecular dynamics simulations (MDS) are used to a chosen set of compounds is also known [33]. Therefore, it is of interest to report screening of potential ligands from the ZINC database with RAC1B using molecular docking and dynamics simulation analysis.

## Materials and Methods:

## Collection of datasets:

A dataset of five experimentally validated RAC1B inhibitors-ZINC69391, NSC23766, EHop-016, MBQ-167, and EHT-1864-was compiled for pharmacophore modeling and virtual screening [34]. The biological activities of these compounds are as follows- ZINC69391, a specific Rac1 inhibitor, interferes with Rac1-GEF interaction by masking Trp56 residue on the Rac1 surface. ZINC69391 can block Rac1 activation in breast cancer cells by interference of Rac1-Tiam1 interaction [35]. In breast cancer cells, inhibition of Rac activity by NSC23766 was shown to induce G1 cell cycle arrest or apoptosis. NSC23766 was designed to prevent the activation of Rac1 by binding to the region where several GEFs interact with Rac1, thus inhibiting its activation. This mode of action was recently confirmed via analysis of the crystal structure of Rac1 with NSC23766 [36]. EHop-016 is an effective Rac-specific inhibitor at micromolar concentrations. EHop-016 substantially inhibits Vav2 interaction with Rac, Rac-activated PAK1, lamellipodia formation and cell migration. EHop-016 proves to be a valuable, more potent probe for the study of Rac-regulated cellular processes [37]. MBQ-167 inhibits metastatic breast cancer cell migration and MBQ-168 promotes loss of cancer cell polarity to result in disorganization of the actin cytoskeleton and detachment from the substratum [38]. The small molecule EHT1864 binds Rac1/1b/2/3 and promotes loss of guanine nucleotide association, locking Rac in an inactive conformation and inhibiting GTPase activity and engagement of downstream effectors [39].

## Pharmacophore modelling and screening:

Pharmacophore-based virtual screening was performed using ZINCPharmer (http://zincpharmer.csb.pitt.edu/), an online interface integrated with the ZINC database and powered by Pharmer search technology [40]. A pharmacophore model was generated based on the five reference inhibitors (ZINC69391, NSC23766, EHop-016, MBQ-167, and EHT-1864), using their common interaction features such as hydrogen bond donors/acceptors, hydrophobic centers, and aromatic rings. A pharmacophore describes the spatial arrangement of the essential features of an interaction. The software can provide information on 3D chemical structures covering the hydrophobicity, electrophilicity, donor and hydrogen bond acceptor regions [41]. The molecules have been assigned biologically relevant protonation states and are annotated with properties such as molecular weight calculated LogP and number of rotatable bonds. Each molecule in the library contains vendor and purchasing information and is ready for docking using several popular docking programs. Within certain limits, the molecules are prepared in multiple protonation states and multiple automatic forms. In one format, multiple conformations are available for the molecules. This database is available for free download (http://zinc.docking.org) in several common file formats including SMILES, mol2, 3D SDF and DOCK flexible format [42].

## Protein and ligand preparation:

The crystal structure of RAC1B used in this study was retrieved from the Protein Data Bank (PDB) (https://www.rcsb.org) with the PDB ID: 1RYF, resolved at 1.75 Å and the structure 1RYF consists of two identical proteins labelled A and B chains [43]. Proteins were prepared for docking in such a way that all the non-receptor atoms such as water, ions, etc. were removed. Kollman charges were assigned. Solvation parameters were added to the final macromolecule structure using the Addsol utility of AutoDock. The known RAC1B inhibitor EHop-016 (PubChem CID: 3080557) was retrieved from the PubChem database (https://pubchem.ncbi.nlm.nih.gov) in SMILES format [44]. The SMILES string was converted into a 3D PDB structure using Open Babel GUI (v3.1.1). Similarly, all ligands obtained from the virtual screening (in SDF format) were converted into PDB format using Open Babel. Each ligand was then prepared using AutoDock Tools, where Gasteiger charges were applied, non-polar hydrogens merged, aromatic carbons detected, rotatable bonds assigned, and TORSDOF set. All ligands were saved in PDBQT format for compatibility with AutoDock 4.2.6 [45, 46].

## Active site prediction:

The active site of the protein was detected using the site map module of Schrodinger software based on site score all the possible sites were clustered in all the regions of the target protein.

## Molecular docking:

Molecular docking was performed to predict the binding affinity and interaction profiles of the screened ligands with the RAC1B protein. The docking studies were carried out using AutoDock Vina v1.2.3, with input files prepared through AutoDock Tools (ADT) v1.5.7. The protein structure (chain A of PDB ID: 1RYF) was pre-processed as described earlier and saved in PDBQT format. Ligands were also converted into PDBQT format following geometry optimization and charge assignment. A grid box was defined to cover the GTP-binding region of RAC1B with the following parameters: Center coordinates: X = -2.444, Y = 69.965, Z = 35.521 Box dimensions: X = 20 Å, Y = 20 Å, Z = 20 Å. These grid settings ensured comprehensive coverage of the known active site region. Docking was performed with an exhaustiveness parameter of 8, ensuring a balance between computational efficiency and pose accuracy. The energy range was set to 3 kcal/mol, and a fixed random seed was used for reproducibility. The pose with the lowest binding free energy was selected for further analysis. Post-docking interaction analysis was carried out using BIOVIA Discovery Studio Visualizer 2021. Hydrogen bonding, hydrophobic interactions, Π-Π stacking, and other non-covalent contacts between the protein and ligand were evaluated, and 2D interaction maps were generated to visualize binding site interactions.

## ADME properties:

The pharmacokinetic properties (Absorption, Distribution, Metabolism, and Excretion - ADME) of the five top-screened compounds (ZINC5277811, ZINC16485670, ZINC20530618, ZINC29990274, ZINC35288817) along with the reference inhibitor EHop-016 were evaluated using the SwissADME online tool (http://www.swissadme.ch/) [47]. The SMILES representations of the compounds were retrieved from the PubChem and ZINC databases and submitted to SwissADME for analysis. SwissADME is a free, user-friendly web-based computational tool employed for the assessment of pharmacokinetics and medicinal chemistry friendliness of small molecules.

## Molecular dynamics simulation:

Molecular dynamics (MD) simulations were performed using the Desmond Molecular Dynamics package (Schrödinger Release 2022-4) with the OPLS_2005 force field. Protein-ligand complexes from molecular docking were selected based on binding energy and interaction profile. Each complex was placed in a cubic simulation box with a 10 Å buffer distance from the edges to prevent interactions with periodic images. The system was solvated using the TIP3P explicit water model, and 0.15 M NaCl was added to neutralize the system and mimic physiological conditions [48]. Energy minimization was performed using the steepest descent algorithm for a maximum of 5000 steps with a convergence threshold of 25 kcal/mol/Å. Following minimization, the system underwent equilibration using Desmond's default NPT ensemble relaxation protocol, which includes both NVT (constant volume and temperature) and NPT (constant pressure and temperature) stages. The production MD run was conducted for 100 nanoseconds using an NPT ensemble at a temperature of 300 K (maintained using the Nose-Hoover thermostat) and pressure of 1 atm (controlled using the Martyna-Tobias-Klein barostat). A time step of 2 femtoseconds was used, and trajectory snapshots were saved every 100 ps for post-simulation analysis [49].

## Results and Discussion:

The best 5 pharmacophore features were selected for screening. The screening was performed through the zinc database, with resulting 5000 hits. Based on the lowest RMSD, 1000 compounds were selected and they were further filtered through molecular docking. The active site was predicted using a sitemap and the high-ranked clusters were selected based on the site score. The Site Score and druggability score (D-Score) of RAC1B is 0.883 and 0.824 respectively and the residues of the binding site are ASP11, GLY12, ALA13, VAL14, GLY15, LYS16, THR17, CYS18, LEU19, PHE28, TYR32, ILE33, PRO34, THR35, PHE37, ASP38, TYR40, ASP57, THR58, ALA59, THR134, LYS135, ASP137, LEU138, CYS176, SER177, ALA178, LEU179. The computed parameters of the binding site are given in Table 1 (see PDF). The best compounds were identified finally through the best binding energy and the newly identified molecules involved in the hydrogen bond interaction residues as well as the hydrophobic interactions. So the hydrogen bond interaction indicates that it has good specificity towards the target and the hydrogen bond indicates the ligand has a good binding affinity towards the target protein with the key amino acids residues binding CYS18, PHE28, ILE33, THR17, LYS16 and PRO29 which make hydrophobic interaction with the screened compounds and reported one make ALA178, LEU138, LEU179, LYS135, CYS18, PHE28 and Hydrogen bond interaction with the key amino acids residues are ALA13, ALA178, LYS16, LYS135, LEU179, GLY15, THR17 it is given in the Table 2 (see PDF) and Figure 1 (see PDF).

The ADME properties were calculated via the Swiss ADME web server (Swiss Institute of Bioinformatics, Switzerland) given in Table 3 (see PDF) [50]. The pharmacological properties of the selected compounds were calculated by Qikprop. Qikprop was run for a set of newly identified compounds. In the Qikprop module, the ADME properties of the identified compounds explored were highly suited for drug formulation. According to that, the MW (molecular weight) of the molecule should be ranges between than 130-725 g/mol, the solvent accessible surface area (SASA) between 7-200.0, the rotatable bonds between 0-15, the partition coefficient (LogP) should be ≤5 and the number of hydrogen bond donors (0-6) and acceptors should be (2-20), predicted apparent Caco- cell permeability of all compounds is (>0.9), intestinal absorption cut-offs between (>80% high/<25%poor), the BBB permeability ranges between (-3.0-1.0) and CNS permeability between (-2 to +2) respectively [51]. All of these qualities, together with molecular flexibility, are thought to be important drivers of oral bioavailability. If these cut-off values correspond to the respective drug then a drug could be orally absorbed into the human body. The obtained ADME attributes are within the suggested ranges. The compounds which are not satisfying the ADME properties are eliminated from the study. MW - Molecular weight of the molecule = 130.0-725 Da. DHB - Number of donor hydrogen bonds = 0.0-6.0. AHB - Number of hydrogen bond acceptors = 2.0-20.0. RB - No.of Rotatable Bonds = 0.0-15.0PSA - Surface area of polar nitrogen and oxygen atoms and carbonyl carbon atoms = 7-200QP log Po/w - Predicted octanol/water partition coefficient = <5QPPCaco - Predicted apparent Caco-2 cell permeability in nm/sec. = >0.9QP log BB Permeability - Predicted brain/blood partition coefficient = -3.0 to 1.2 CNS Permeability = -2 to +2% HOA - Percentage of human oral absorption = >80% is high, <25% is low.

Desmond was used to analyze the molecular dynamics simulation. To gain insight into protein-ligand interaction stability of the best-docked protein-ligand complex, MDS for 100 ns have been performed using Desmond suite in Schrodinger [52]. The Root Mean Square Deviation (RMSD) of the protein-ligand complex shows that the screened compounds Zinc5277811, Zinc16485670, Zinc20530618, Zinc29990274 and Zinc35288817 are initially fluctuating and it continues till 30 to 40 ns. After that, all the protein-ligand complexes are getting stable throughout the simulations time. But the reported compound EHop_016 fluctuated throughout the simulation time given in (Figure 2 - see PDF). The Root Mean Square Fluctuation (RMSF) data depicts that there were fewer fluctuations observed with the active site residues of all screen compounds (Figure 3 - see PDF). Additionally, the hydrogen bond interaction diagram shows all the screened compounds maintain the hydrogen bond interaction consistently even though there was a loss of hydrogen bond interaction in the middle of the simulations. But the reported compound failed to maintain the hydrogen bond interaction during the simulation illustrated in Figure 4 (see PDF) & 5 (see PDF). Finally, the hydrogen bond and hydrophobic bond residue contribution for all the screened compounds and the reported compound are given in Figure 6 (see PDF). Overall, molecular dynamics simulation data clearly indicates that in all aspects the screened compounds have better protein-ligand binding stability than the reported compound EHop_016.

## Conclusion:

The five lead compounds ZINC5277811, ZINC16485670, ZINC20530618, ZINC29990274, and ZINC3528881 showed optimal binding affinity, stability compared to the known inhibitor EHop-016 with RAC1B protein target for further consideration.

## List of abbreviations:

ADME = Absorption, Distribution, Metabolism, Excretion

MDS = Molecular Dynamics Simulation

RMSD = Root Mean Square Deviation

RMSF = Root Mean Square Fluctuation

HBA = Hydrogen Bond Acceptor

HBD = Hydrogen Bond Donor

HOA = Percentage of human oral absorption

PSA = Polar Surface Area

RB = Rotatable Bonds

MW = Molecular Weight

DHB = Donor Hydrogen Bonds

AHB = Acceptors Hydrogen Bonds

BB = Brain/Blood

CNS = Central Nervous System

PL = Protein Ligand

HB = Hydrogen Bond

## Data availability statement:

The associated author can provide the data supporting the results that have been reported and they will send it by mail upon request.

## References

[1] Singh R (2024). Sci Rep..

[2] https://www.who.int/news-room/.

[3] Siegel RL (2022). CA Cancer J Clin..

[4] https://www.ncbi.nlm.nih.gov/books/NBK13081/.

[5] Azim HA (2014). J Thorac Dis..

[6] Ferlay J (2021). Int J Cancer..

[7] Vostakolaei FA (2011). Eur J Public Health..

[8] Tfayli A (2010). J Oncol..

[9] Sung H (2021). CA Cancer J Clin..

[10] Williams AS (2018). J Am Board Fam Med..

[11] Chen F (2023). Oncogene..

[12] Fiegen D (2004). J Biol Chem..

[13] Engers R (2007). Endocr Relat Cancer..

[14] Devreotes P (2015). Cold Spring Harb Perspect Biol..

[15] Porter AP (2016). Small GTPases..

[16] Montalvo-Ortiz BL (2012). J Biol Chem..

[17] Hofbauer SW (2014). Blood..

[18] Hein AL (2016). Oncogene..

[19] Cardama GA (2018). Crit Rev Oncol Hematol..

[20] Sun J (2021). Oncogene..

[21] Marei H (2017). Small GTPases..

[22] Gudiño V (2021). Nat Commun..

[23] Azios NG (2007). Neoplasia..

[24] Cabrera M (2017). Oncotarget..

[25] Sugihara K (1998). Oncogene..

[26] Olabi S (2018). Breast Cancer Res..

[27] Tang Y (2008). Front Biosci..

[28] Wells CM (2004). J Cell Sci..

[29] Zhou J (2020). Mol Biomed..

[30] Humphries B (2020). Cells..

[31] Verma K (2024). Bioinformation..

[32] Schnelzer A (2000). Oncogene..

[33] Pece S (2010). Cell..

[34] Yoshida T (2010). Mol Cancer Ther..

[35] Maldonado MDM (2018). Cancer Res..

[36] Cardama GA (2014). Anticancer Agents Med Chem..

[37] Rozenveld-Geugien M (2007). Exp Hematol..

[38] Martin H (2013). J Clin Invest..

[39] Medina JI (2022). Cancer Res Commun..

[40] Onesto C (2008). Methods Enzymol..

[41] Koes DR (2012). Nucleic Acids Res..

[42] Chandra I (2021). SAR QSAR Environ Res..

[43] Schapira M (2003). J Med Chem..

[44] Hatami S (2022). Res Pharm Sci..

[45] Singh S (2016). J Biomol Struct Dyn..

[46] Reddy KK (2014). Mol Biosyst..

[47] Szklarczyk D (2015). Nucleic Acids Res..

[48] Daina A (2017). Sci Rep..

[49] Kumar S (2020). J Chem Inf Model..

[50] Murthy PK (2018). J Mol Struct..

[51] https://www.schrodinger.com/platform/products/qikprop/.

[52] Terefe EM (2022). Bioinform Biol Insights..

